# Dysregulation of miR-23b-5p promotes cell proliferation via targeting FOXM1 in hepatocellular carcinoma

**DOI:** 10.1038/s41420-021-00440-0

**Published:** 2021-03-15

**Authors:** Xinchen Yang, Shikun Yang, Jinhua Song, Wenjie Yang, Yang Ji, Feng Zhang, Jianhua Rao

**Affiliations:** Hepatobiliary Center, The First Affiliated Hospital of Nanjing Medical University; Key Laboratory of Liver Transplantation, Chinese Academy of Medical Sciences, NHC Key Laboratory of Liver Transplantation, Nanjing, China

**Keywords:** Oncogenes, Targeted therapies

## Abstract

Growing evidence demonstrates that MicroRNAs (miRNAs) play an essential role in contributing to tumor development and progression. However, the underlying role and mechanisms of miR-23b-5p in hepatocellular carcinoma (HCC) formation remain unclear. Our study showed that miR-23b-5p was downregulated in the HCC tissues and cell lines, and lower expression of miR-23b-5p was associated with more severe tumor size and poorer survival. Gain- or loss-of-function assays demonstrated that miR-23b-5p induced G0/G1 cell cycle arrest and inhibited cell proliferation both in vitro and in vivo. qRT-PCR, western blot and luciferase assays verified that Mammalian transcription factor Forkhead Box M1 (FOXM1), upregulated in HCC specimens, was negatively correlated with miR-23b-5p expression and acted as a direct downstream target of miR-23b-5p. In addition, miR-23b-5p could regulate cyclin D1 and c-MYC expression by directly targeting FOXM1. Further study revealed that restoration of FOXM1 neutralized the cell cycle arrest and cell proliferation inhibition caused by miR-23b-5p. Taken together, our findings suggest that miR-23b-5p acted as a tumor suppressor role in HCC progression by targeting FOXM1 and may serve as a potential novel biomarker for HCC diagnosis and prognosis.

## Introduction

Hepatocellular carcinoma (HCC) is a common malignancy and the third leading cause of cancer-related mortality worldwide, particularly in China with high prevalence of hepatitis B virus (HBV)/hepatitis C virus(HCV) infection^1,2^. In recent years, despite the great progression in HCC treatment, the 5-year survival rate of patients with HCCs remains unsatisfactory due to the high frequency of recurrence and metastasis^3,4^. Given the asymptomatic nature in the early stages, most HCC patients are diagnosed at advanced stages. Therefore, it is critical to elucidate the mechanisms underlying the HCC progression and explore the effective therapeutic strategies to improve the diagnosis and treatment of HCC^5^.

MicroRNAs are endogenous, single-stranded, small non-coding RNAs regularly 21 to 24 nucleotides in length. By binding to the 3′-untranslated region (3′UTR) of the target messenger RNA (mRNA), they could modulate gene expression at post-transcriptionally level and affect multiple essential biological processes, such as cell proliferation, movement, metastasis, differentiation, apoptosis and death^6–8^. Previous researches have shown the role of miR-23b-5p in a variety of pathological and physiological processes. Oumarou et al.^7^ found that miR-23b-5p was involved in promotion of cardiac hypertrophy and dysfunction via activating HMGB2 signaling pathway. You et al.^8^ confirmed the role of miR-23b-5p in thermogenic program by regulating brown adipogenesis. In addition, increasing number of researches have illustrated the suppressive function of miR-23b-5p in the progression of malignant tumors^9,10^. However, the specific biological function and mechanisms of miR-23b-5p in HCC has yet to be determined.

Mammalian transcription factor Forkhead Box M1 (FOXM1) belongs to the extensive family of Forkhead transcription factors, characterized by a conserved protein domain called Forkhead or winged-helix domain, which known as a key regulator in tumor growth and cell-cycle regulation^11^. FOXM1 could exert oncogenic functions by regulating multiple downstream targets in human malignant tumors^12,13^, such as CCND1 and c-MYC^14,15^. Increasing studies have shown that FOXM1 is extensively overexpressed in most human malignancies, including HCC, breast cancer and lung cancer, and its increased expression may serve as a biomarker and indicate the poor outcome of patients^16–19^. Meanwhile, recent investigation suggested that miRNAs could be involved in the functions of FOXM1^20,21^. However, little is known about the functional link between miR-23b-5p and FOXM1 in HCC.

In this study, we verified the downregulated miR-23b-5p expression in HCC tissues and cell lines, and found that HCC patients with higher miR-23b-5p expression showed a better overall survival. Furthermore, CCK-8 assay, EdU assay and colony formation assays demonstrated that overexpressed miR-23b-5p could inhibit cell proliferation. Mechanistically, miR-23b-5p could induce HCC cell cycle arrest at the G1 phase by directly binding to the 3’UTR of FOXM1. These findings indicated that miR-23b-5p functioned as a tumor suppressor miRNA in the progression of liver cancer by decreasing the expression of FOXM1, and miR-23b-5p may serve as a novel diagnostic and prognostic biomarker for HCC.

## Results

### miR-23b-5p expression is downregulated in HCC tissues and cell lines

**Fig. 1:**
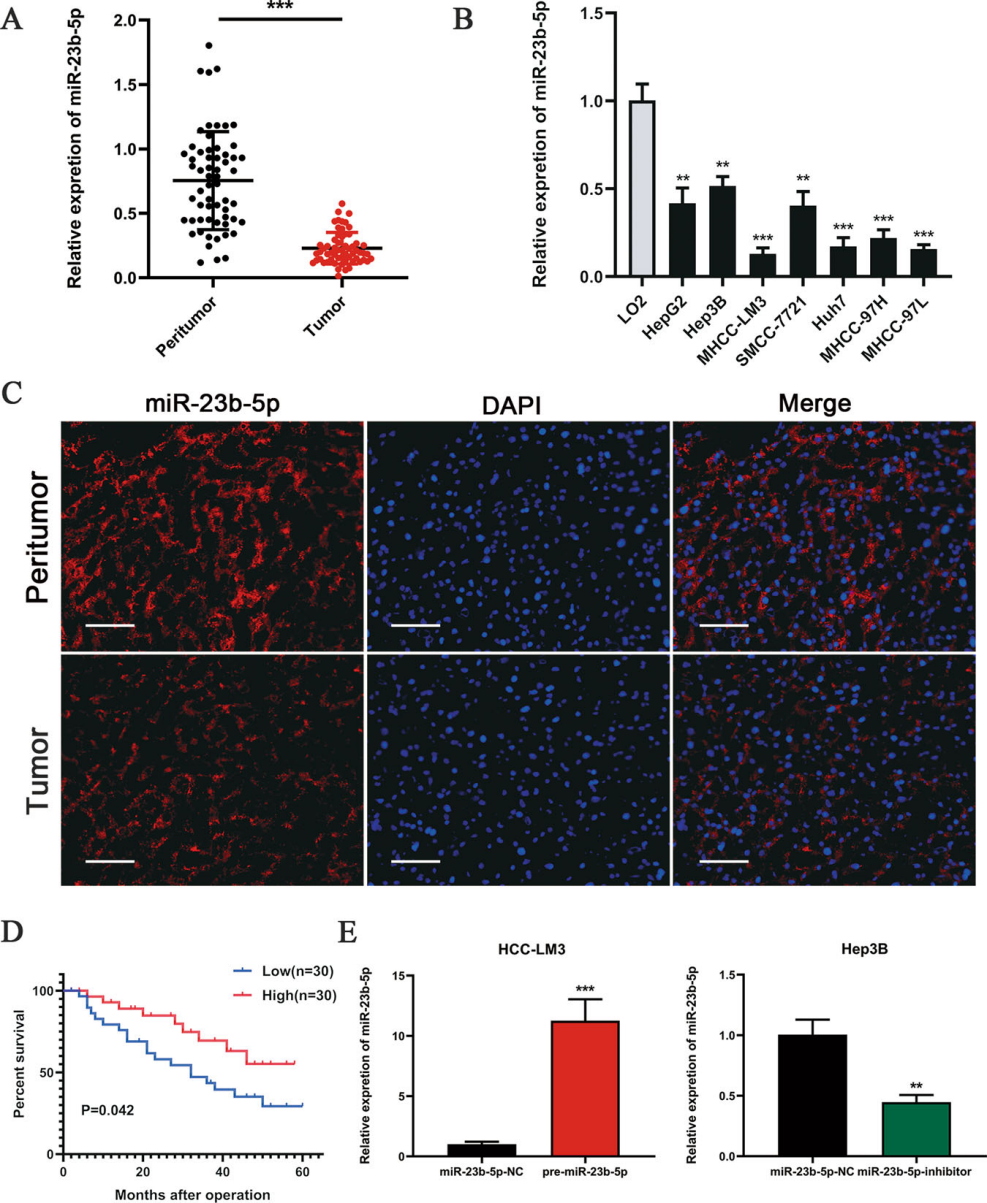
miR-23b-5p is downregulated in HCC tissues and cell lines. **A** The mRNA levels of miR-23b-5p in 60-paired human HCC and adjacent normal tissues were tested by qRT PCR. **B** The mRNA levels of miR-23b-5p expression in HCC cell lines and normal LO2 cells. **C** Expression of miR-23b-5p in HCC and adjacent normal tissues was measured by FISH assay (scale bars, 100 μm). **D** Kaplan–Meier analysis for overall survival of 60 patients with HCC resection according to the miR-23b-5p expression. **E** The HCC-LM3 and Hep3B cells were transfected with lentivirus overexpressing miR-23b-5p (pre-miR-23b-5p) or lentivirus with short hairpin RNA targeting miR-23b-5p (miR-23b-5p-inhibitor). Cells transfected with empty lentiviral vectors served as a negative control (NC). The miR-23b-5p expression levels were analyzed by qRT-PCR. All data are presented as the mean ± S.E.M. (*P < 0.05, **P < 0.01, and ***P < 0.001).

To identify whether miR-23b-5p was dysregulated in HCC tissues, we investigated miR-23b-5p expression in 60-paired HCC tissues and adjacent peritumor tissues by qRT-PCR. We observed lower miR-23b-5p expression level in tumor tissues compared with adjacent nontumor tissues (Fig. 1A). Consistent with this result, the analysis of TCGA database also showed downregulated level of miR-23b-5p in HCC tissues (Supplementary Fig. S1A). In addition, compared to immortalized human hepatocyte LO2 cells, miR-23b-5p expression in HCC cells (Hep3B, HepG2, HCCLM3, SMMC-7721, Huh-7, MHCC-97H and MHCC-97L) was significantly decreased (Fig. 1B). Moreover, we empolyed four paired HCC and peritumoral tissues for FISH analysis of miR-23b-5p and further confirmed the lower miR-23b-5p expression in HCC tissues (Fig. 1C). To investigate the clinicopathological features of miR-23b-5p in HCC, patients involved in this study were divided into high or low-group based on median value. As shown in Table 1, lower miR-23b-5p expression was significantly associated with larger tumor size (*P* < 0.05). In addition, we performed Kaplan–Meier analysis on these 60 HCC patients, and revealed that lower miR-23b-5p expression was correlated with poorer overall survival (Fig. 1D, *P* < 0.01).

**Table 1:** Association between miR-23b-5p expression and clinicopathologic features of patients with hepatocellular carcinoma.

		miR-23b-5p		
Characteristics	Number	Low	High	*P*-value
All cases	60	30	30	
Age(years)				0.592
≤60	22	12	10	
>60	38	18	20	
Gender				0.584
Female	20	11	9	
Male	40	19	21	
Liver cirrhosis				0.766
No	15	7	8	
Yes	45	23	22	
HBsAg status				0.573
Negative	18	8	10	
Positive	42	22	20	
α-fetoprotein(ng/ml)				0.197
≤20	31	13	18	
>20	29	17	12	
Tumor size(cm)				0.038^a^
≤5	28	10	18	
>5	32	20	12	
Tumor multiplicity				0.795
Single	33	17	16	
Multiple	27	13	14	
Edmondson grade				0.195
I–II	33	19	14	
III–IV	27	11	16	
Tumor-node-metastasis stage				0.197
I–II	29	17	12	
III–IV	31	13	18	

^a^Indicates *P* < 0.05.

### miR-23b-5p inhibits HCC cell growth and induces cell cycle arrest

**Fig. 2:**
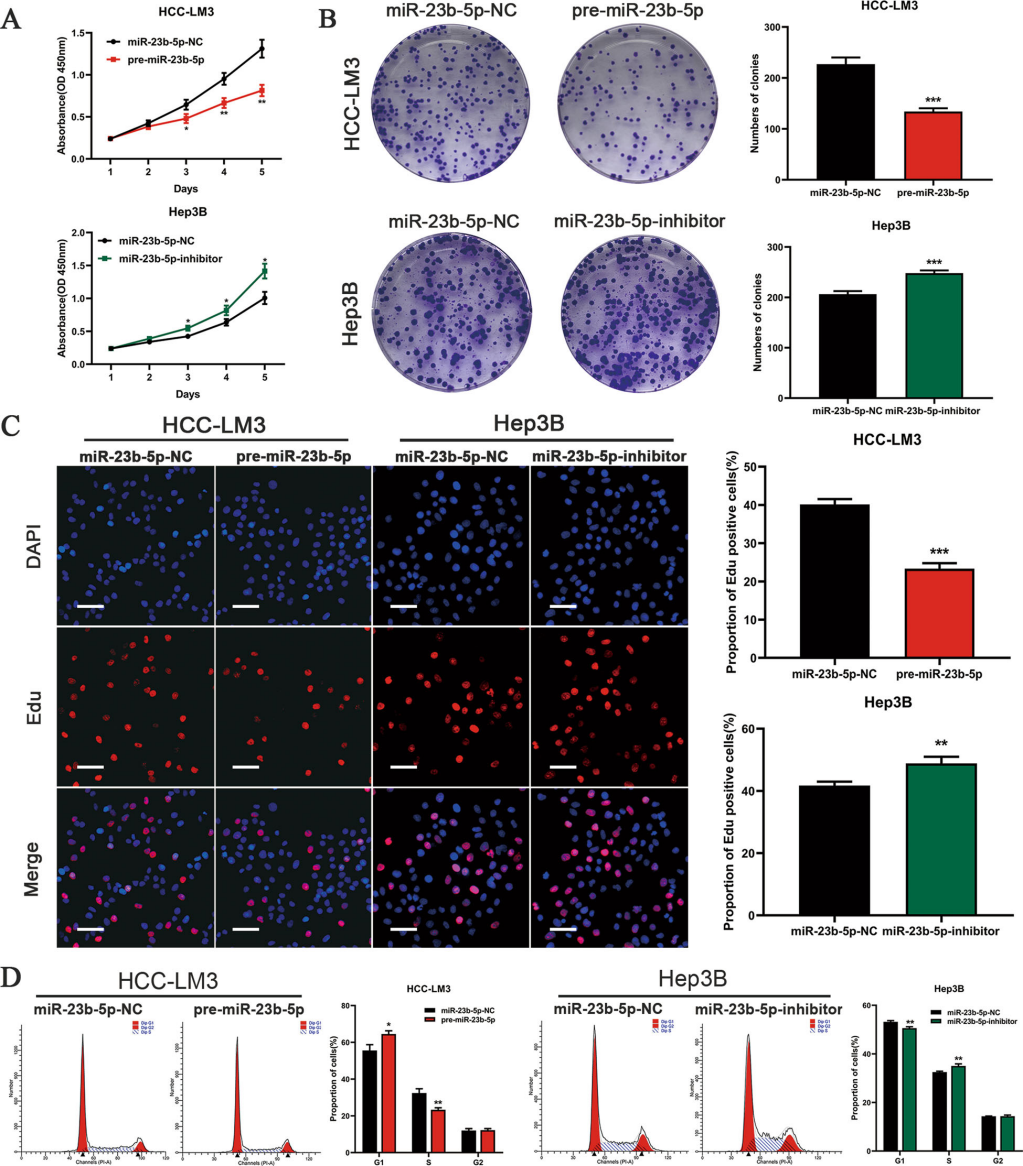
miR-23b-5p suppresses HCC cell proliferation and induces cell cycle arrest. **A** CCK-8 assays in the HCC-LM3 and Hep3B cells overexpressing or silencing miR-23b-5p. **B** Colony formation assays in overexpressing or silencing miR-23b-5p HCC-LM3 and Hep3B cells. **C** 5-ethynyl-2’-deoxyuridine (EdU) incorporation assays were performed to explore the cell proliferation in miR-23b-5p-overexpressing or -silencing HCC-LM3 and Hep3B cells (scale bars, 50 μm). **D** The flow cytometry results showed the contribution of miR-23b-5p overexpressing or silencing in HCC-LM3 and Hep3B cells to cell cycle. All data are presented as the mean ± S.E.M. (*P < 0.05, **P < 0.01, and ***P < 0.001).

As shown in Fig. 1B, the HCC cell line HCC-LM3 and Hep3B exhibited the lowest and highest expression level of miR-23b-5p respectively. We transfected pre-miR-23b-5p into HCC-LM3 cells and transfected miR-23b-5p inhibitor into Hep3B cells respectively. qRT-PCR was performed to confirm the transfection efficiency. HCC-LM3 cells treated with pre-miR-23b-5p presented a marked increase in the miR-23b-5p expression level, while Hep3B cells transfected with miR-23b-5p inhibitor notably inhibited the miR-23b-5p expression level compared to negative control group (Fig. 1E). To evaluate the function of miR-23b-5p in cell proliferation, CCK-8 assays, EdU assays and colony formation assays were conducted. In the CCK8 assays, compared with the control groups, we found that upregulation of miR-23b-5p significantly inhibited the proliferation of HCC-LM3 cells, whereas downregulation of miR-23b-5p significantly promoted the growth of Hep3B cells (Fig. 2A). Meanwhile, ectopic expression of miR-23b-5p suppressed the colony formation ability of HCC-LM3 cells. In contrast, knockdown of miR-23b-5p promoted colony formation in Hep3B cells (Fig. 2B). Consistent with these results, EdU assays showed that stable overexpression of miR-23b-5p decreased the numbers of EdU-positive nuclei of HCC-LM3 cells compared with the controls, while stable knockdown of miR-23b-5p increased the numbers of EdU-positive nuclei (Fig. 2C). As miR-23b-5p notably inhibited HCC cell proliferation, we aimed to ascertain whether miR-23b-5p could regulate the cell cycle of HCC cells. As determined by flow cytometry, the effects of miR-23b-5p dysregulation on the cell cycle were showed in Fig. 2D. We observed that overexpression of miR-23b-5p in the HCC-LM3 cells presented an obvious increase in G0/G1 phase, while knockdown of miR-23b-5p in the Hep3B cells led to a marked decrease in G0/G1 phase compared with the control group. These data demonstrated that overexpressed miR-23b-5p inhibited HCC cell growth and induced G0/G1 cell cycle arrest.

### FOXM1 is upregulated in human HCC tissues and cell lines and correlated with poor survive

**Fig. 3:**
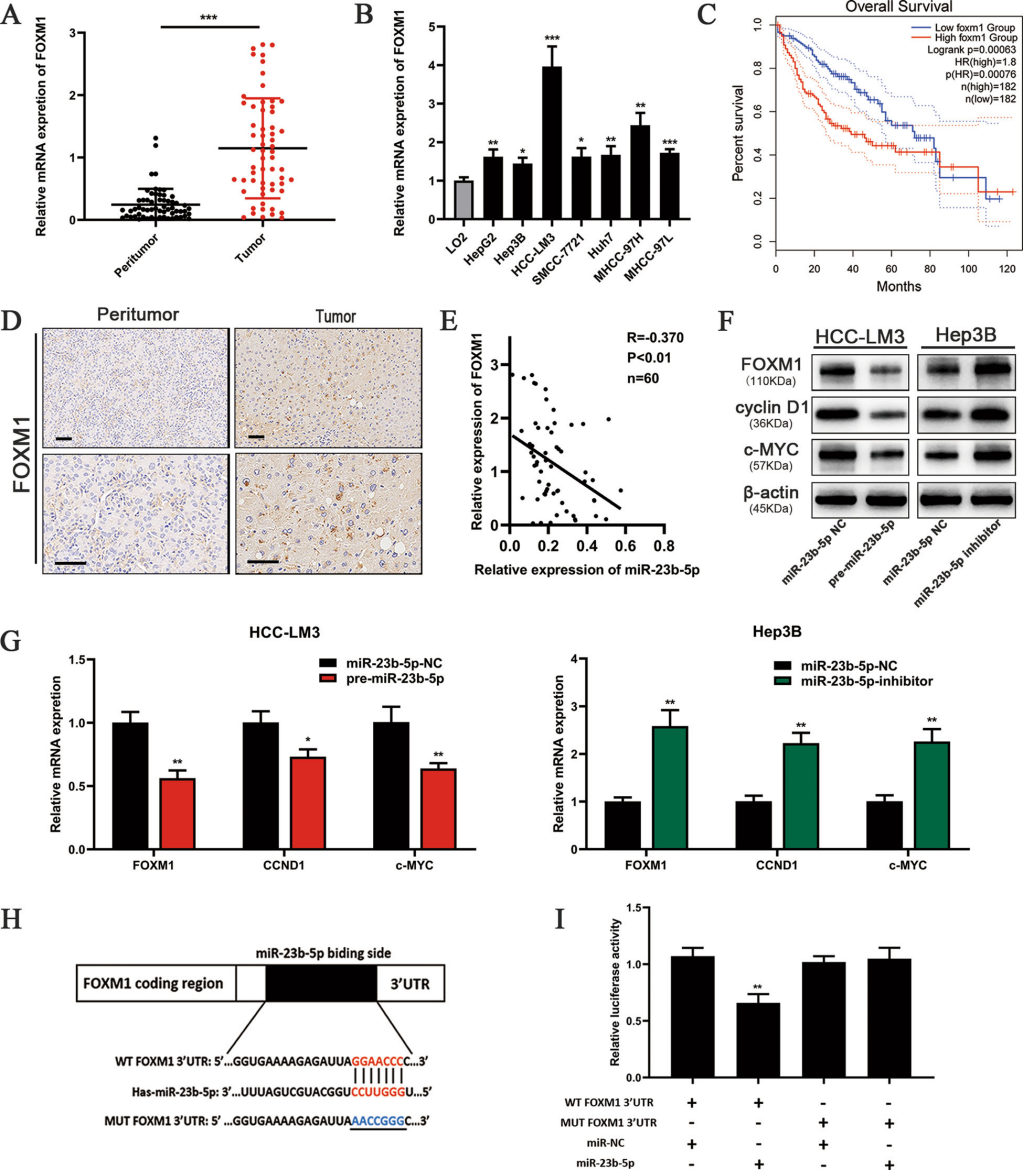
FOXM1 is upregulated in human HCC tissues and cell lines and miR-23b-5p directly targets FOXM1 in HCC cells. **A**, **B** Expression levels of FOXM1 in the HCC tissues and cell lines were detected by qRT-PCR. **C** Kaplan–Meier analysis for overall survival of patients with HCC according to FOXM1 expression level in all patients (GEPIA database). **D** Representative immunostaining images of FOXM1 in HCC tissues and adjacent normal tissues (scale bars, 50 μm). **E** Spearman correlation analysis confirmed the correlations between the FOXM1 mRNA and miR-23b-5p expression levels in the 60 HCC samples (*r* = −0.370, *P* < 0.01). **F**, **G** Western blotting and qRT-PCR analysis of the expression levels of FOXM1, CCND1, and c-MYC in the HCC-LM3 cells transfected with miR-23b-5p-NC or pre-miR-23b-5p and the Hep3B cells transfected with miR-23b-5p-NC or miR-23b-5p-inhibitor. **H** The predicted miR-23b-5p targeting sequence in the FOXM1 3′-UTR (WT FOXM1 3′ UTR). The target sequences of the FOXM1 3′ UTR were mutated (MUT FOXM1 3′ UTR). **I** Dual-luciferase reporter assay of the cells transfected with WT FOXM1 3′ UTR or MUT FOXM1 3′ UTR reported together with 40 nM of the miR-23b-5p mimic or negative control oligoribonucleotides. All data are presented as the mean ± S.E.M. (*P < 0.05, **P < 0.01, and ***P < 0.001).

As miR-23b-5p induced cell proliferation inhibition, we utilized bioinformatic databases (TargetScan and miRWalk) to predict the potential downstream target of miR-23b-5p. Among them we particularly interested in FOXM1, since FOXM1 had been reported to play a central role in the control of cell proliferation and was widely recognized as a key player in cell cycle progression^16,18^. Previous studies revealed that FOXM1 was highly expressed in HCC tissues and closely associated with poor prognosis^22–24^. We analyzed FOXM1 expression from TCGA database (Supplementary Fig. S1B) and in 60-paired HCC tissues and adjacent normal tissues by qRT-PCR (Fig. 3A). Compared to adjacent normal tissues, FOXM1 expression was significantly lower in HCC tissues and this result was further verified by immunohistochemistry (Fig. 3D). Consistent with the above data, mRNA level of FOXM1 was also lower in HCC cells compared to LO2 cell (Fig. 3B). In addition, Kaplan–Meier analysis indicated that higher FOXM1 expression was correlated with a remarkable shorter overall survival time (Fig. 3C).

### miR-23b-5p directly targets FOXM1 in HCC cells

We have already determined the miR-23b-5p and FOXM1 expression in different HCC cell lines via qRT-PCR (Figs. 1B and 3B). The results showed that miR-23b-5p was expressed at a low level, while FOXM1 expression levels were increased compared to those observed in LO2 cells. In addition, bioinformatic analysis of TCGA database also showed downregulated miR-23b-5p and upregulated FOXM1 level in HCC (Supplementary Fig. S1A, B). Furthermore, we compared FOXM1 and miR-23b-5p expression level in 60 pairs of HCC tissues and found that they were negatively correlated (Fig. 3E). Western blot and qRT-PCR demonstrated that upregulation of miR-23b-5p resulted in decreased FOXM1 transcription and protein levels, while downregulated miR-23b-5p led to the opposite results (Fig. 3F–G). Luciferase reporter assay was used to further confirm that FOXM1 was a direct target of miR-23b-5p. Wild-type (WT) or mutant (MUT) FOXM1–3′ UTR were inserted into a luciferase reporter vector. The results demonstrated that miR-23b-5p suppressed luciferase activity of the WT-FOXM1–3′ UTR, whereas the luciferase activity of the MUT-FOXM1–3′ UTR was almost unchanged (Fig. 3H, I). These data suggested miR-23b-5p could directly and negatively regulate FOXM1.

### Restoration of FOXM1 reverses the effects of miR-23b-5p on HCC cells

**Fig. 4:**
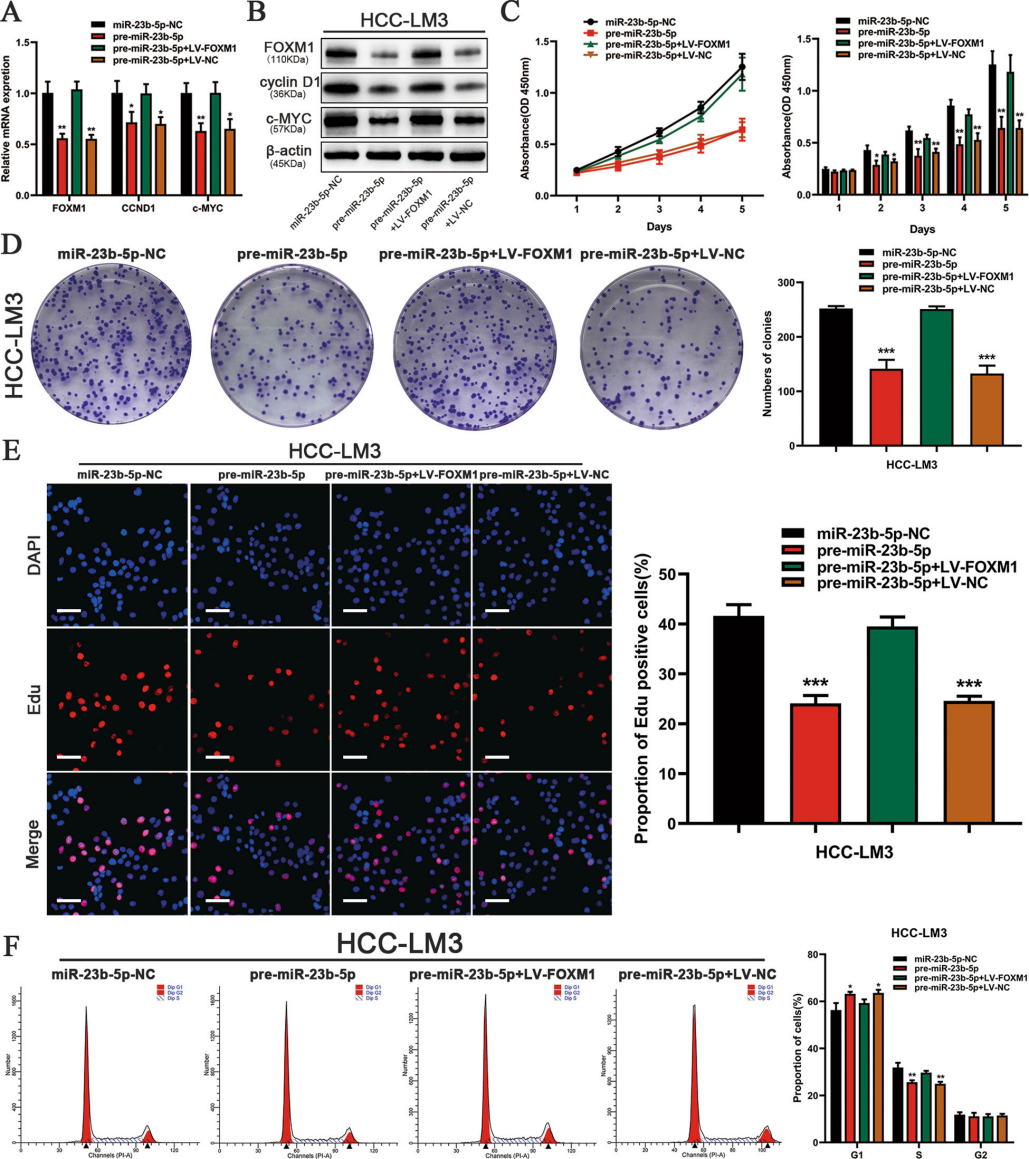
Restoration of FOXM1 were performed to confirm that FOXM1 is the functional target of miR-23b-5p. **A**, **B** The expression of FOXM1 and its downstream targets, CCND1 and c-MYC, verified by qRT PCR and western blot in the HCC-LM3 cells transfected with the miR-23b-5p overexpression lentivirus (pre-miR-23b-5p), FOXM1 overexpression lentivirus (LV-FOXM1) and negative control lentivirus (LV-NC). **C** CCK-8 assay, **D** colony formation assay, and **E** 5-ethynyl-2’-deoxyuridine (EdU) incorporation assay were used to verify the proliferation (scale bars, 50 μm) and **F** flow cytometry were utilized for cell cycle test of miR-23b-5p-NC, pre-miR-23b-5p, pre-miR-23b-5p plus LV-FOXM1, and pre-miR-23b-5p plus LV-NC in the HCC-LM3 cells. All data are presented as the mean ± S.E.M. (*P < 0.05, **P < 0.01, and ***P < 0.001).

To further illustrate that miR-23b-5p inhibited the proliferation of HCC cells by regulating FOXM1. HCC-LM3 cells were transfected with pre-miR-23b-5p lentivirus. After 72 h, the cells were transfected with LV-FOXM1. FOXM1 upregulation was corroborated by qRT-PCR and western blot (Fig. 4A, B). Through CCK-8, EdU and colony formation assays, we confirmed that FOXM1 restoration reversed the proliferation-inhibiting effect of miR-23b-5p (Fig. 4C–E). Moreover, flow cytometry analysis was conducted to investigate whether miR-23b-5p affected the cell cycle through the modulation of FOXM1 in HCC cells. It turned out that high expression of FOXM1 could abolish cell cycle arrest caused by upregulated miR-23b-5p in the HCC-LM3 cells (Fig. 4F). These findings indicated that restoration of FOXM1 could neutralize the influence of miR-23b-5p on HCC cell lines.

### miR-23b-5p regulated CCND1/c-MYC by targeting FOXM1

In the preceding studies, we showed that miR-23b-5p could affect the cell cycle of HCC cancer cells. Therefore, we examined miR-23b-5p contribution to cell cycle by flow cytometry, and the results showed that miR-23b-5p could increase the ratio of cells in G0/G1 period and miR-23b-5p inhibitor had the opposite effect (Fig. 2D). In addition, restoration of FOXM1 could neutralize the influence of miR-23b-5p. As we all know, cyclin D1 and c-myc were crucial for the regulation of cell proliferation and the cell cycle and were important targets of FOXM1^14,15^. We have confirmed that miR-23b-5p could directly target FOXM1. Thus, we wondered whether miR-23b-5p could regulate the expression levels of CCND1 and c-MYC via FOXM1. We evaluated the expression levels of CCND1 and c-MYC by qRT-PCR and western blot after transfection with pre-miR-23b-5p and miR-23b-5p-inhibitor lentivirus. HCC-LM3 cells overexpressing miR-23b-5p demonstrated a clear downregulation of CCND1 and c-MYC mRNA and protein expression level. Meanwhile, Hep3B cells with downregulated expression of miR-23b-5p showed a moderate increase in cyclin D1 and c-myc protein expression and marked upregulated mRNA expression level compared to the negative control (Fig. 3F, G). Furthermore, we performed rescue experiments to verify the effects of restoration of FOXM1. As Fig. 4A, B indicated, decreased expression levels of CCND1 and c-MYC were restored when transfected with LV-FOXM1. These findings demonstrated that miR-23b-5p could regulate CCND1/c-MYC expression levels by targeting FOXM1.

### miR-23b-5p inhibits xenograft tumor formation

**Fig. 5:**
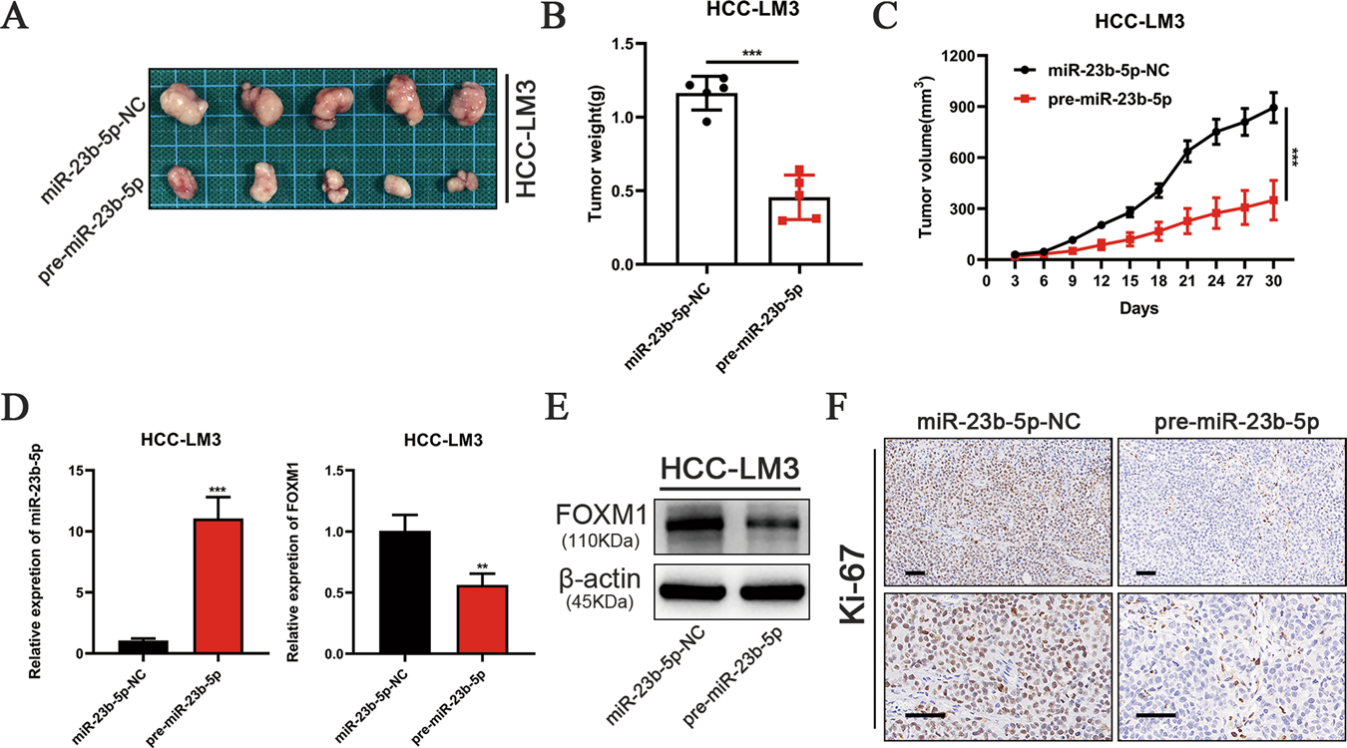
miR-23b-5p suppresses xenograft tumor growth in vivo. **A**–**C** Photographs of tumors derived from the different groups of nude mice transfected with HCC-LM3-miR-23b-5p-NC and HCC-LM3-pre-miR-23b-5p cells. The tumors were measured for volume and average weight. **D** The miR-23b-5p and FOXM1expression levels of xenografts were analyzed by qRT-PCR. **E** The FOXM1 expression levels of xenografts were detected by western blot. **F** Ki67 staining of tumor tissues mice injected with HCC-LM3-miR-23b-5p-NC and HCC-LM3-pre-miR-23b-5p cells (scale bars, 50μm). All data are presented as the mean ± S.E.M. (*P < 0.05, **P < 0.01, and ***P < 0.001).

To further investigate whether miR-23b-5p inhibited proliferation of HCC cells in vivo, HCC-LM3 cells transfected with miR-23b-5p-NC and pre-miR-23b-5p lentivirus were injected into nude mice. During tumor formation, the volume of tumor was recorded every 3 days, and the mice were euthanized 30 days later. The tumors generated from the HCC-LM3-pre-miR-23b-5p group were marked smaller and weight less than the NC group (Fig. 5A–C). Moreover, the results of qRT-PCR and western blot revealed that FOXM1 expression was significantly downregulated in the pre-miR-23b-5p group compared to the control group both in mRNA and protein level (Fig. 5D, E). In addition, immunohistochemistry showed that miR-23b-5p overexpression decreased the number of Ki67-positive cells (Fig. 5F). These results verified that miR-23b-5p suppressed tumor growth of HCC in vivo.

## Discussion and conclusion

Numerous studies have confirmed that aberrantly expressed miRNAs could affect the proliferation and metastasis of HCC cells. In recent years, therapies based on miRNAs have been used in several preclinical models, including liver cancer^25^. Thus, the researches on miRNAs played important roles in providing new strategies for HCC diagnosis, prognosis, and therapy. Therefore, it is necessary to illuminate the relevant mechanisms between miRNAs and HCC.

Recently, the functional role of miR-23b-5p in malignant tumors has been proposed. Hu et al.^9^ found that miR-23b-5p was downregulated and acted as a sponge target of lncRNA to enhance the progression of lung adenocarcinoma. Farina et al.^26^ showed miR-23b-5p could influence prostate tumorigenesis and a bioinformatic analysis from Shan et al.^10^ revealed that miR-23b-5p may play an important role in the progression of hepatocellular carcinoma. Meanwhile, Warnecke-Eberz et al.^27^ isolated the exosomes from serum of patients with adenocarcinoma of the esophagus and showed that miR-23b-5p was merely detected in exosomes. Consistent with this result, Barrera-Ramirez et al.^28^ detected the exosomes from marrow-derived mesenchymal stromal cells in patients with acute myeloid leukemia and found that miR-23b-5p had a downregulated expression level in exosome. These data suggested that miR-23b-5p played an essential role in the progression of carcinoma formation. Nevertheless, the exact biological functions and molecular mechanisms of miR-23b-5p underlying HCC formation remain unknown. In this study, we demonstrated that the expression level of miR-23b-5p in HCC tissues and cell lines are downregulated. According to the data from 60 HCC patients, we found that patients with low expression level of miR-23b-5p had a larger tumor size and poorer outcomes compared to those with high miR-23b-5p. Investigation in vivo and in vitro both confirmed the function of miR-23b-5p in cell proliferation and cell cycle distribution. We found that upregulation of miR-23b-5p could suppress cell growth and induced G0/G1 cell cycle arrest, whereas knockdown of miR-23b-5p showed opposite results, it made a great increase in proliferation and downregulated the population of cells in G0/G1 phase of the cell cycle. Taken together, our results suggested that miR-23b-5p functioned as a tumor suppressor with whose downregulation the progression of HCC could be promoted.

It is well known that miRNAs could typically bind and destabilize target mRNAs to exert its functions in regulating gene expression. Thus, we selected the target gene of miR-23b-5p with the help of biological tools and we focused on FOXM1 because of its strong biological function. FOXM1 was recognized as a potent oncogene accountable for poor prognosis in various carcinomas and acted a pivotal role in regulating DNA damage response, oxidative stress, drug resistance and progression of tumor growth^29–33^. FOXM1 was widely verified as a key player in cell cycle progression and could regulate a network of proliferation associated genes that are involved in mitotic spindle assembly, G1/S and G2/M transition and chromosome segregation^16,17,34–36^. In this study, we demonstrated that FOXM1 expression was increased and negatively correlated with miR-23b-5p expression in HCC tissues. Then, to validate whether miR-23b-5p could directly bind to the 3’ UTRs of FOXM1, the luciferase activity assay was conducted. Furthermore, restoration experiments of FOXM1 was performed, we found that upregulated FOXM1 partially reversed the miR-23b-5p induced proliferation-inhibiting effect and cell cycle arrest in HCC cells. These data showed that miR-23b-5p could suppress HCC cell proliferation and induced G0/G1 cell cycle arrest via FOXM1.

To further investigate the mechanism of miR-23b-5p impacting on cell proliferation, we put our eyes on the network of proliferation associated genes regulated by FOXM1. Wierstra et al.^15^ reported that FOXM1 could transactivate the c-myc promoter by binding directly to its TATA-boxes and Wang et al.^14^ showed that FoxM1 also activated transcription of cyclin D1 promoter. Meanwhile, FOXM1 was involved in several significant signaling pathways such as the Wnt/β-catenin pathway and interacts with its critical members, thereby contributing to signal transduction^37^. Zhang et al.^38^ verified that FOXM1 could interact with β-catenin, and promote β-catenin nuclear localization. When the FOXM1-β-catenin complex enters the nucleus, it further forms a transcriptional complex with T-cell factor (TCF) or lymphoid enhancer factor (LEF) to activate the expression of Wnt target genes including c-MYC and Cyclin D1^38^. The c-MYC was well known as an oncogene contributes to the genesis of many human cancers^39^. Cyclin D1,belongs to the G1 cyclin family and plays a role in regulating the transition through the G1 phase of the cell cycle^40^. Accumulating studies have reported that Cyclin D1 functioned as an oncogene and its overexpression was associated with a variety of human malignancies, including HCC^41,42^. Thus, We hypothesized that cyclin D1 and c-myc may play an important role in miR-23b-5p impacting on HCC proliferation. In our study, we found the expression level of Cyclin D1 and c-MYC were decreased when miR-23b-5p was upregulated in HCC cells, whereas the downregulated miR-23b-5p group showed the opposite trend by western blot and qRT-PCR. Furthermore, we confirmed that when rescued the FOXM1 expression level in cells the changes caused by dysregulation of miR-23b-5p could be partially reversed.

In conclusion, our findings demonstrated that the expression levels of miR-23b-5p were downregulated in HCC tissues, which was related to poor prognosis of HCC patients. Overexpression of miR-23b-5p significantly reduced HCC proliferation and resulted in G0/G1 cell cycle arrest by targeting FOXM1. Therefore, miR-23b-5p may serve as a potential biomarker for the diagnosis and prognosis of HCC, and provide a targeted approach to HCC therapy.

## Materials and methods

### Tissue samples and cell lines

In this study, 60-paired HCC and adjacent normal tissues from patients who underwent curative hepatectomy at The First Affiliated Hospital of Nanjing Medical University, China were tested. We obtained consent from the patients or their relatives before the collection of specimens. The study was approved by the Ethics Committee of Nanjing Medical University. All human HCC cells lines (Hep3B, HepG2, HCC-LM3, SMMC-7721, Huh-7, MHCC-97H and MHCC-97L) and the human normal liver cell line (LO2) were purchased from the Chinese Academy of Sciences (Shanghai, China). All cells were cultured in DMEM medium (Gibco, NY, USA) supplemented with 10% fetal bovine serum (Gibco), 100 U/ml penicillin (Gibco), and 100 mg/ml streptomycin (Gibco) at 37 °C in a humidified cell incubator in a 5% CO_2_ atmosphere.

### RNA isolation and quantitative real-time PCR (qRT-PCR) analysis

We extracted total RNA from HCC tissues and cells using TRIzol reagent (Invitrogen, USA) following the manufacturer’s instructions. Complementary DNA (cDNA) was reverse transcribed using a PrimeScript RT Reagent kit with gDNA Eraser (TaKaRa, RR047A) according to the manufacturer’s instructions. The miR-23b-5p primers were synthesized by RiboBio (Guangzhou, China). The qRT-PCR procedure used to detect the miR-23b-5p level was: cycle 1, 95 °C for 2 min; cycle 2 through 40, 95 °C for 30 s, 60 °C for 35 s, and fluorescence signal was detected at the end of each cycle. The expression level of the specific transcripts was normalized to internal controls (β-actin or U6). The primers used in cyclin D1, myc and FOXM1 mRNA detection were shown as follows. Cyclin D1 forward: 5′-AACTACCTGGACCGCTTCCT-3′, reverse: 5′-CCACTTGAGCTTGTTCACCA-3′. MYC forward: 5′-TCAAGAGGCGAACACACAAC-3′, reverse: 5′-GGCCTTTCATTGTTTTCCA-3′. FOXM1 forward: 5′-CGTCGGCCACTGATTCTCAAA−3′, reverse: 5′- GGCAGGGGATCTCTTAGGTTC−3′. β-actin forward: 5′-TGGCACCCAG CACAATGAA-3′, reverse: 5′-CTAAGTCATAGTCCGCCTAGAAGCA-3′. Detection of each sample was repeated 3 times and the results were calculated using the 2^-ΔΔCT^ method.

### Establishment of stably transfected cells

We purchased commercially available LV-hsa-miR-23b-5p-mimic (pre-miR-23b-5p), LV-hsa-miR-23b-5p-inhibitor (miR-23b-5p-inhibitor), LV-has-miR-23b-5p-NC, LV-FOXM1, and LV-FOXM1-NC constructed in lentiviral vectors from GenePharma (Shanghai, China). After infecting the lentiviruses with HCC-LM3 and Hep3B cells, puromycin (Sigma-Aldrich, USA) was used to select cells according to protocols.

### Cell counting Kit-8 assay

The effect of miR-23b-5p on cell growth was evaluated with a Cell Counting Kit-8 (CCK-8, Dojindo,Tokyo, Japan). Transfected cells were incubated in 96-well plates at a density of 1 × 10^3^ cells per well and cultured as previously described.10ul of CCK-8 solution was mixed with 100ul serum-free medium and added to each well every 24 h. After 2 h of incubation, we detected the absorbance at 450 nm in a microplate reader. The cells were cultured for 5 days.

### Colony formation assay

We seeded 500 cells per well into 6-well plates and cultured as described before to evaluate the colony formation ability. We observed colonies every day and after the cells were cultured for 10 days. The medium was removed and washed by PBS for 3 times. The cells were fixed with ethyl alcohol for 30 s and stained with 1% crystal violet for 15 min. The numbers of colonies were counted manually.

### 5-ethynyl-2′-deoxyuridine (Edu) proliferation assay

EdU proliferation assay (Ribobio, China) was used to evaluate the cell proliferation ability. Cells in the logarithmic growth phase were seeded in 24-well plates at 10,000 cells per well for 1 day. On the following day, cells were incubated with EdU for 2 h. Then, cells were neutralized with 2 mg/ml glycine and permeabilized in 0.5% Triton X-100 for 20 min. After extensive washing with PBS, cells were incubated with Apollo staining reaction buffer for 30 min. Subsequently, the nuclei of cells were stained with DAPI for 15 min, and the EdU incorporation rate was analyzed with a fluorescence microscope.

### Flow cytometric analysis

To analyze cell cycle, sufficient amounts of cells were collected and added with 70% prechilled ethanol for 2 h overnight. On the next day, cells were resuspended in 500 μl of PBS and then stained with 500 μl propidium iodide (PI) (Vazyme, Nanjing, China) for 30 min. The stained cells were analyzed by a BD FACSCanto II (BD Biosciences, USA) flow cytometer. The percentage of the cells in G1, S, and G2/M phase was analyzed using ModFit software.

### Western blot analysis

Total protein was isolated from HCC tissues, cell lines and mouse tumors. BCA kit (Beyotime) was used to measure the protein concentration according to the manufacturer’s instructions. The extracted protein was separated by a 10% SDS–PAGE based on their molecular weight and transferred to a polyvinylidene fluoride (PVDF) membrane (Bio-Rad, CA, USA). After blocking with 5% nonfat dry milk for 2 h, the membranes were incubated with the appropriate primary antibody overnight at 4 °C. Antibodies including rabbit anti-FOXM1 polyclonal antibody, anti-cyclinD1, anti-c-myc were used, and β-actin was used as an internal control. All antibody were purchased from Cell Signaling Technology, Danvers, MA, USA. Membranes were then incubated with HRP-conjugated anti-rabbit IgG (1:2000) for 2 h at room temperature and then washed with TBST buffer three times. Protein expression levels were detected by ECL Plus (EMD Millipore, Billarica, MA, USA).

### Fluorescence in situ hybridization (FISH)

We performed FISH to evaluate miR-23b-5p expression in HCC tissues and matched normal tissues. The mature human miR-23b-5p sequence is 3′- UUUAGUCGUACGGUCCUUGGGU-5′. We purchased the miR-23b-5p antisense oligonucleotide probes from Servicebio (Wuhan, China). The FISH assay was conducted as previously described^43^.

### Immunohistochemical staining

The HCC tissues were fixed in 4% paraformaldehyde, embedded in paraffin and cut into 4-μm sections. After blocking endogenous peroxides and proteins, the sections were incubated with the primary antibody FOXM1and Ki-67 (Cell Signaling Technology, Danvers, MA, USA). On the following day, the sections were incubated with secondary antibody at 37 °C for 1 h, incubated with 3,3’-diaminobenzidine solution for 3 min and then counterstained with hematoxylin. The tumor sections were examined in a blinded manner.

### Dual-luciferase reporter assay

The potential binding sites of miR-23b-5p in the FOXM1 3’ UTR were predicted by TargetScan7.2 and miRWalk. The wild-type 3’-UTR sequences or the mutated sequences of FOXM1 were inserted into the pmir-GLO-promoter vector (Promega, Madison, USA). We seeded cells into a 24-well plate the day before transfection. Then, luciferase reporter plasmids were cotransfected with the miR-23b-5p mimic or negative control using Lipofectamine 3000 (Invitrogen). Luciferase activities were measured with the Dual Luciferase Reporter Assay System (Promega, USA).

### Bioinformatic analysis

Clinical information about hepatocellular carcinoma (50 HCC tissues and paired 50 normal hepatic tissues) was obtained from TCGA database (The Cancer Genome Atlas, https://cancergenome.nih.gov). Differentially expressed miRNAs and mRNAs were filtered using Bioconductor R version 3.4.1 (https://www.r-project.org/) and “edgeR” program package. The screening thresholds were | log_2_(fold change) | > 1 and adjusted *P*-value < 0.05.

### Tumor xenograft in animals

For tumor growth assay, ten nude mice (aged 4 weeks) were obtained from the Animal Model Institute of Nanjing University (Nanjing, China). Nude mice were randomly divided into 2 groups (*n* = 5 per group). HCC-LM3-NC and HCC-LM3-pre-miR-23b-5p stable cells (2 × 10^6^ cells) in 100 μl PBS were injected to nude mice by subcutaneous injection. We recorded the tumor size every 3 days and euthanized the mice 30 days later.

### Statistical analysis

Data are expressed as mean ± standard deviation from at least three experiments. The *χ*^2^ test was used to analyze the association of miR-23b-5p expression with clinicopathological features. Independent *t*-tests were used to compare two groups’ differences. A paired *t*-test was used to analyze miR-23b-5p and FOXM1 mRNA levels in tissue samples. Correlations between miR-23b-5p and FOXM1 were determined by Pearson’s correlation analysis. Statistical analyses were performed using GraphPad software and SPSS, *P < 0.05, **P < 0.01, and ***P < 0.001 were defined as statistically significant.

## Supplementary information

Figure S1
