# Supplementary figures and images for: Dysregulation of miR-23b-5p promotes cell proliferation via targeting FOXM1 in hepatocellular carcinoma
Source: Cell Death Discov. 2021 Mar 15;7:47. doi: 10.1038/s41420-021-00440-0 (PMC7960996; doi:10.1038/s41420-021-00440-0)

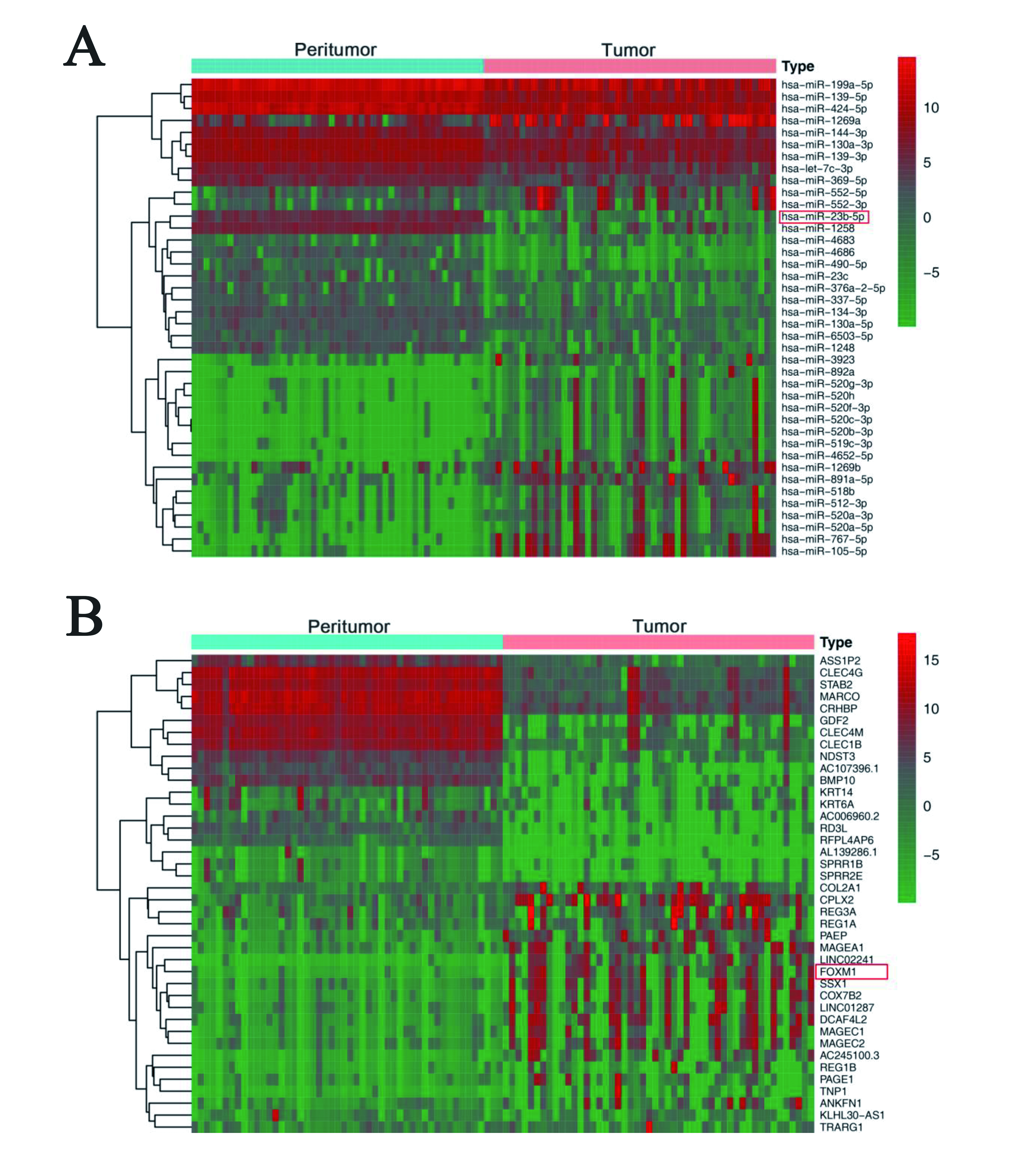

Supplement: Supplementary file 1 — Figure S1 [file 41420_2021_440_MOESM1_ESM.tif]
